# In vivo quantification of rolling and adhered leukocytes in human sepsis

**DOI:** 10.1186/s13054-018-2173-z

**Published:** 2018-09-30

**Authors:** Bjorn K. Fabian-Jessing, Michael J. Massey, Michael R. Filbin, Peter C. Hou, Henry E. Wang, Hans Kirkegaard, Donald M. Yealy, William C. Aird, John A. Kellum, Derek C. Angus, Nathan I. Shapiro, on behalf of the ProCESS Investigators

**Affiliations:** 1 Department of Emergency Medicine, Beth Israel Deaconess Medical Center, Harvard Medical School, 1 Deaconess Road, CC2-W, Boston, MA 02215 USA; 2 Research Center for Emergency Medicine, Aarhus University Hospital, Aarhus, Denmark; 3 Department of Emergency Medicine, Massachusetts General Hospital, Harvard Medical School, Boston, MA USA; 4 Department of Emergency Medicine, Brigham and Women’s Hospital, Harvard Medical School, Boston, MA USA; 5 Department of Emergency Medicine, University of Alabama at Birmingham, Birmingham, AL USA; 6 Department of Emergency Medicine, University of Pittsburgh, Pittsburgh, PA USA; 7 Center for Vascular Biology and Department of Medicine, Beth Israel Deaconess Medical Center, Harvard Medical School, Boston, MA USA; 8 Department of Critical Care Medicine, University of Pittsburgh, Pittsburgh, PA USA

**Keywords:** Sepsis, Septic shock, Microcirculation, Leukocyte, Sidestream dark field, SDF

## Abstract

**Background:**

The use of in vivo videomicroscopy at the bedside has demonstrated microcirculatory flow disturbances in sepsis. The ability of in vivo videomicroscopy to detect changes in the prevalence of rolling and adhered leukocytes that occur in sepsis is not well-described in humans. We sought to (1) develop methodology for accessing and quantifying sublingual leukocyte rolling and adherence with sidestream dark field (SDF) imaging; (2) compare the number of rolling and adhered leukocytes between patients with septic shock and non-infected controls; and (3) compare the number of rolling and adhered leukocytes between survivors and non-survivors of septic shock.

**Methods:**

We included adult (age > 18 years) patients in the emergency department presenting with septic shock prospectively enrolled in the ProCESS trial. We recruited comparison non-infected patients as emergency department controls. Using a SDF videomicroscope, we obtained image sequences from the sublingual mucosa, quantifying rolling and adhered leukocytes per 1 mm × 1 mm visual field in a standardized 3-s clip. We report data as median and interquartile range and depicted as box plots. We compared groups using the Mann-Whitney U test, considering a *p* value < 0.05 significant.

**Results:**

We included a total of 64 patients with septic shock and 32 non-infected controls. The median number of adhered leukocytes per field in the sepsis group was 1.0 (IQR 0–3.5) compared to 0 (0–0) in the non-infected group (*p* < 0.001). The median number of rolling leukocytes was 26 (10.3–42) in the sepsis group and 9.8 (4.8–17.3) in the non-infected group (*p* < 0.001) per field. Among the patients with sepsis (*n* = 64), there was an increased number of adhered leukocytes in non-survivors compared to survivors (3.0 (1–5.5) vs. 1.0 (0–3.0)) (*p* < 0.05); however, there was no difference in rolling leukocytes (35 (20–48) vs. 26 (10–41)) (*p* = 0.31).

**Conclusions:**

Our results demonstrated a higher number of rolling and adhered leukocytes in patients with septic shock when compared to non-infected controls, and an increased number of adhered leukocytes in non-survivors.

**Trial registration:**

ClinicalTrials.gov, NCT00793442; Registered on 19 November 2008

PG0GM076659 (US NIH Grant/Contract). First submitted 18 July 2007. First posted 2 August 2007.

**Electronic supplementary material:**

The online version of this article (10.1186/s13054-018-2173-z) contains supplementary material, which is available to authorized users.

## Background

Endothelial cell dysfunction and microcirculatory flow alterations are elements of sepsis pathophysiology. As a result of the host immune response to infection, there is up-regulation of endothelial and leukocyte adhesion molecules, which initiates leukocyte recruitment and activation [1]. Eventually, adhesive bindings will overcome post-capillary shear stress forces resulting in slowing and rolling, tethering, firm adherence and, ultimately, transmigration (extravasation) of leukocytes [2].

In the experimental setting, many have observed leukocyte-endothelial cell interactions and microcirculatory flow disturbances in sepsis [3, 4], primarily using intravital microscopy in different animal models. Similarly, others using the same tool have observed increased numbers of rolling and adhered leukocytes on the endothelial surface of different organs during experimental sepsis [3, 5]. However, intravital microscopy (as it requires transillumination of the organ of interest and fluorescent dyes) is not readily suitable for human studies. Recent advances in imaging techniques such as sidestream dark field (SDF) imaging have enabled direct in vivo bedside visualization of the human sublingual microcirculation. Multiple SDF studies show a reduction in the capillary density of the microvasculature and a decrease in the adequacy of erythrocyte flow are associated with adverse outcomes in critical illnesses, especially sepsis [6, 7]. Furthermore, microcirculatory disturbances may occur in the presence of normalized macrocirculation (and macrocirculatory disturbances may occur with intact microcirculation), and microcirculatory changes are independently prognostic of adverse outcomes [8, 9].

Since the emphasis of human in vivo studies of the (sublingual) microcirculation has traditionally focused on erythrocyte flow, direct assessment of leukocyte-endothelium interactions in humans is limited [10–12]. Based on the experimental theories and initial human studies, we sought to advance the methodology and study leukocyte rolling and adhesion in sepsis. Accordingly, we aimed (1) to develop methodology for accessing and quantifying sublingual leukocyte activation with SDF imaging; (2) to compare the number of rolling and adhered leukocytes between patients with septic shock and non-infected control patients; and (3) to compare the number of rolling and adhered leukocytes between survivors and non-survivors of septic shock.

## Methods

### Study design and setting

This was a prospective, observational, multicenter study comparing SDF images from emergency department (ED) patients with septic shock enrolled in the Protocolized Care for Early Septic Shock (ProCESS) trial [13] to those from non-infected ED control patients. The ED “control” patients presented with non-infectious, non-shock-related conditions. Patient SDF images (see below for details) within 6 h of enrollment in the ProCESS study protocol were then processed and analyzed offline. The institutional review boards of the University of Pittsburgh, the Beth Israel Deaconess Medical Center, and the participating recruitment sites approved the design. All subjects or their legally authorized representatives provided written informed consent.

### Participants

#### Septic shock cohort

We included adult subjects (age > 18 years) enrolled in this ancillary study of the ProCESS trial [13]. In brief, we enrolled ED patients within 2 h of meeting criteria for septic shock ((1) suspected infection, (2) meeting ≥ 2 criteria for systemic inflammatory response syndrome (SIRS) [14], and (3) evidence of hypoperfusion defined as refractory hypotension (systolic blood pressure < 90 mmHg or required vasopressor use after a minimum 1 L fluid challenge) or serum lactate ≥ 4 mmol/L.) For this ancillary study, we enrolled at six different participating centers.

#### Non-infected control cohort

Control patients were a convenience sample of patients presenting to one of three EDs and having non-infectious complaints that did not meet SIRS criteria and did not have clinical shock.

### SDF image acquisition and selection

The SDF videomicroscope (MicroScan, MicroVision Medical BV, Amsterdam, The Netherlands) is a Food and Drug Administration (FDA)-exempt device, which consists of miniature microscope optics surrounded by light-emitting diodes (LEDs). The LEDs emit a stroboscopic green light with a wavelength of 530 nm, which is absorbed by hemoglobin in red blood cells, and transmitted by leukocytes, which may appear slightly brighter than the illuminated background tissue. SDF imaging allows direct bedside visualization of the sublingual microcirculation. Video images were saved on a laptop computer and uploaded to a server in a de-identified secure fashion.

Using the international consensus criteria [15] and according to the “Microcirculation Image Quality Score” [16], we chose videos for further analysis. We assessed image quality using six factors: illumination (exposure, brightness and contrast), focus (such that most of the vessels appear in the plane of focus), duration (at least 3 s), content (absence of bubbles, saliva, or looped vessels), stability (so that the video can be stabilized effectively without blurring or too much net motion), and pressure (no stopped flow in medium/large vessels or other indicators of iatrogenic pressure). Scores were 0 (good), 1 (acceptable), or 10 (unacceptable). For the assessment of microcirculatory flow, we analyzed further videos with scores < 10; for the quantification of activated leukocytes, we required a quality score ≤ 1 because we found that it was impossible to identify leukocytes in even slightly out-of-focus videos. Up to three videos per patient were selected for further analysis. Videos analysis was performed by investigators unaware of clinical information and not involved in the clinical study protocol or in image acquisition.

### SDF image processing

We used a hybrid method involving video preprocessing and stabilization in MATLAB (Mathworks, Natick, MA, USA), followed by tracing and semi-quantitative flow analysis in a dedicated software package (Automated Vascular Analysis (AVA), MicroVision Medical BV, Amsterdam, The Netherlands). Pre-processing and stabilization involved the following steps: (1) a 3 × 3 median filter is applied to each frame to remove salt-and-pepper noise while preserving relevant image data; (2) inter-frame motion estimate and stabilization using a multiple key-point-feature-based method [17], operating on vessel probability density images [18]; and (3) finally, we enhanced the stabilized images using adaptive histogram equalization [19], then imported pre-processed, stabilized videos into AVA for semi-quantitative analysis.

### Technique for quantification of adhered and rolling leukocytes

For leukocyte quantification, we frame averaged preprocessed and stabilized videos using approximately 1.5 frame Gaussian filter kernel widths. This technique blurs out the column of erythrocytes, plasma gaps and non-activated leukocytes, which constitutes the main blood stream in the SDF images, thus making it easier to identify rolling or adhered leukocytes. The frame-averaged videos were clipped to 90 frames (3 s), so that the middle 90 frames of all videos were selected automatically. Rolling and adhered leukocytes were quantified manually with the assistance of the free software program ImageJ (Rasband, W.S., ImageJ, U. S. National Institutes of Health, Bethesda, MD, USA, http://imagej.nih.gov/ij/, 1997–2014), which allowed playback at different speeds.

**Fig. 1:**
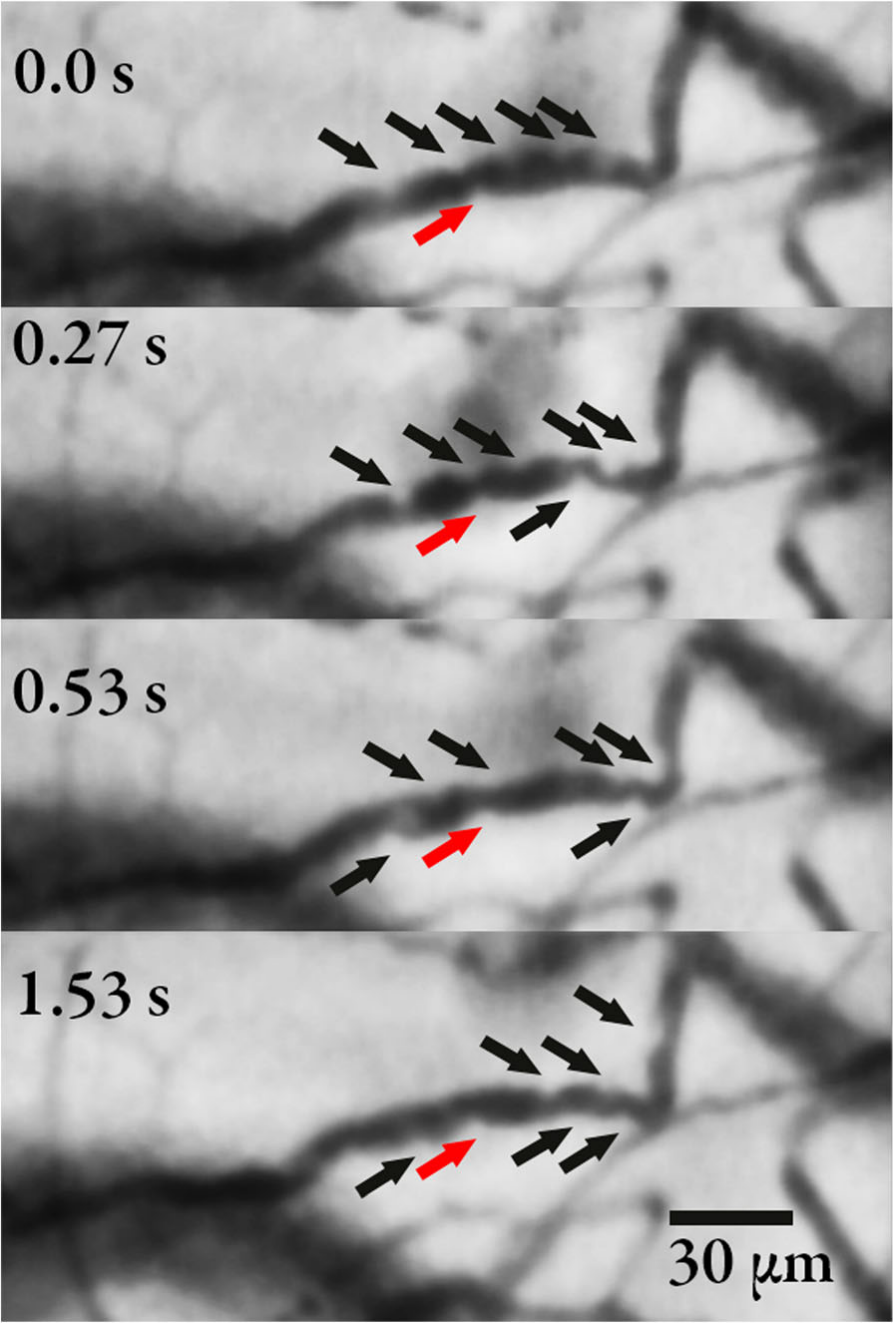
The figure shows the same vessel visualized by sidestream dark field imaging at different time points. The black arrows show rolling leukocytes with a shift towards the right throughout the time course. The red arrow shows an adhered leukocyte

Leukocytes appear as round “defects” in the erythrocyte column, slightly brighter than the background tissue and plasma gaps. In addition, activated (rolling) leukocytes display a characteristic moving pattern along the edges of the erythrocyte column, closer to the surface of the endothelium. Rolling leukocytes were defined as leukocytes moving slower than the main bloodstream. Adhered leukocytes were defined as leukocytes firmly adhered to the vessel lumen such that they were not moving throughout the 3-s clip (Fig. 1 and Additional file 1). Adhered leukocytes that detached from the lumen during the 3-s observation period were counted as rolling. Videos were analyzed consecutively according to a list with videos in a computer-generated random order labeled with computer-generated random numbers. We counted leukocytes in the entire field of view (FOV), in all vessels. To avoid double-counting, all rolling/adhered leukocytes were marked manually in a still picture of the FOV in the respective videos to keep track of the leukocytes during playback. To account for unequal density and distribution of in-focus vessels between videos, we extracted vessel lengths of all in-focus vessels, excluding any out-of-focus vessels. Thus, we calculate the number of activated leukocytes in the FOV and per unit vessel length (number of leukocytes/mm), which is independent of the total vessel density.

### Statistical analysis

Due to the non-normal distribution of the data we report data as median and interquartile range (IQR) and depict data as box plots. We compared groups using the Mann-Whitney U test, considering a *p* value < 0.05 significant.

## Results

We included 64 patients from the ED presenting with septic shock, who were enrolled in the prospective clinical ProCESS trial, and 32 non-infected control ED patients (Table 1).

**Table 1:** Characteristics of the patients

Characteristic	Sepsis (*n* = 64)	Control (*n* = 32)
Past medical history, *n* (%)		
- Diabetes mellitus	21 (32.8)	6 (18.8)
- Chronic obstructive pulmonary disease	20 (31.3)	2 (6.3)
- Liver disease	4 (6.3)	2 (6.3)
- End-stage renal disease	4 (6.3)	2 (6.3)
- AIDS	0 (0)	2 (6.3)
- Cancer	19 (29.7)	5 (15.6)
Metastatic cancer	13 (20.3)	2 (6.3)
- CHF	14 (21.9)	2 (6.3)
- Myocardial infarction	7 (10.9)	4 (12.5)
- Dementia	3 (4.7)	1 (3.1)
Race		
- White	50 (78.1)	22 (68.8)
- Black	5 (7.8)	8 (25.0)
- Asian	6 (9.4)	0 (0.0)
- Other	3 (4.7)	2 (6.3)
Age (SD)	64.8 (13.6)	53.3 (19.0)
Male	34 (53.1)	19 (59.4)
Infection		
- Catheter related	4 (4)	
- CNS	1 (1.61)	
- Endocarditis	1 (1.61)	
- Intra-abdominal	8 (8)	
- None	2 (3.23)	
- Other	3 (4.84)	
- Pneumonia	21 (33.87)	
- Skin and soft tissue	3 (4.84)	
- Unknown	6 (9.68)	
- Urosepsis	13 (20.97)	
Sepsis-related variables		
Leukocyte count (per microliter), mean (SD)	14.6 (10.0)	
Serum lactate (mmol/l), mean (SD)	2.0 (1.8)	
SOFA score, mean (SD)	7.4 (3.5)	
Mortality rate, *n* (%)	11 (17%)	

*CHF* congestive heart failure, *CNS* central nervous system, *SOFA* Sequential Organ Failure Assessment

### Leukocyte rolling and adhesion in sepsis versus control

**Fig. 2:**
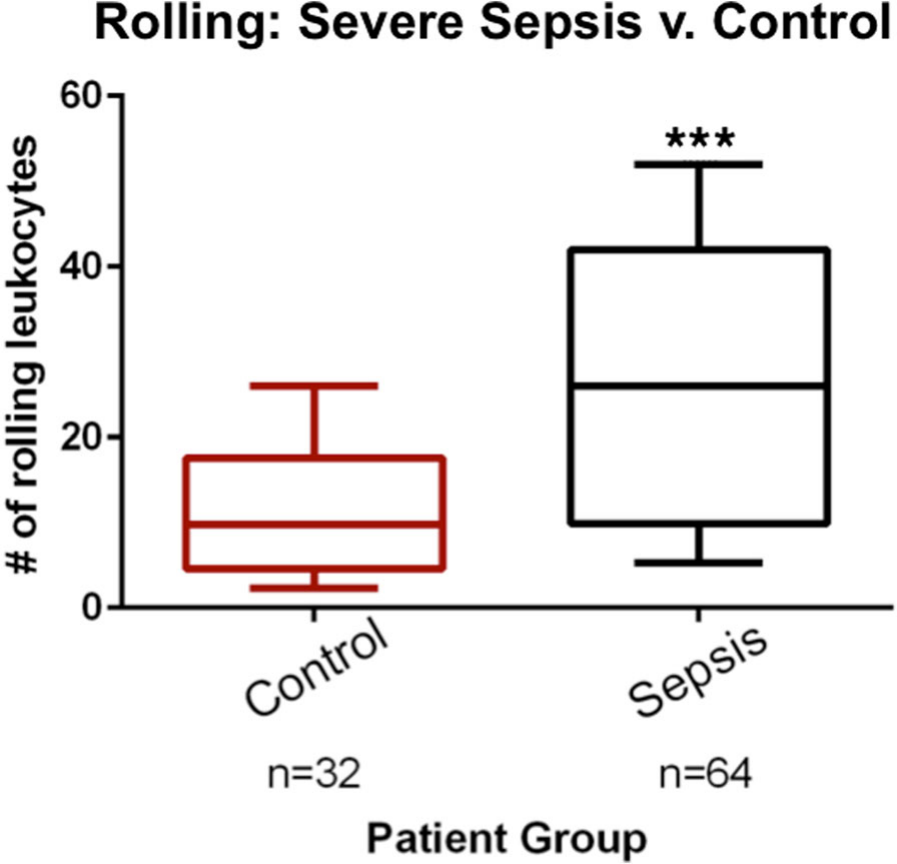
Comparison of adhered leukocytes in patients with sepsis and in controls. The boxes delineate the interquartile range, the median is shown as a line in the middle of the box, and tails show the 95% range; ***p < 0.001

**Fig. 3:**
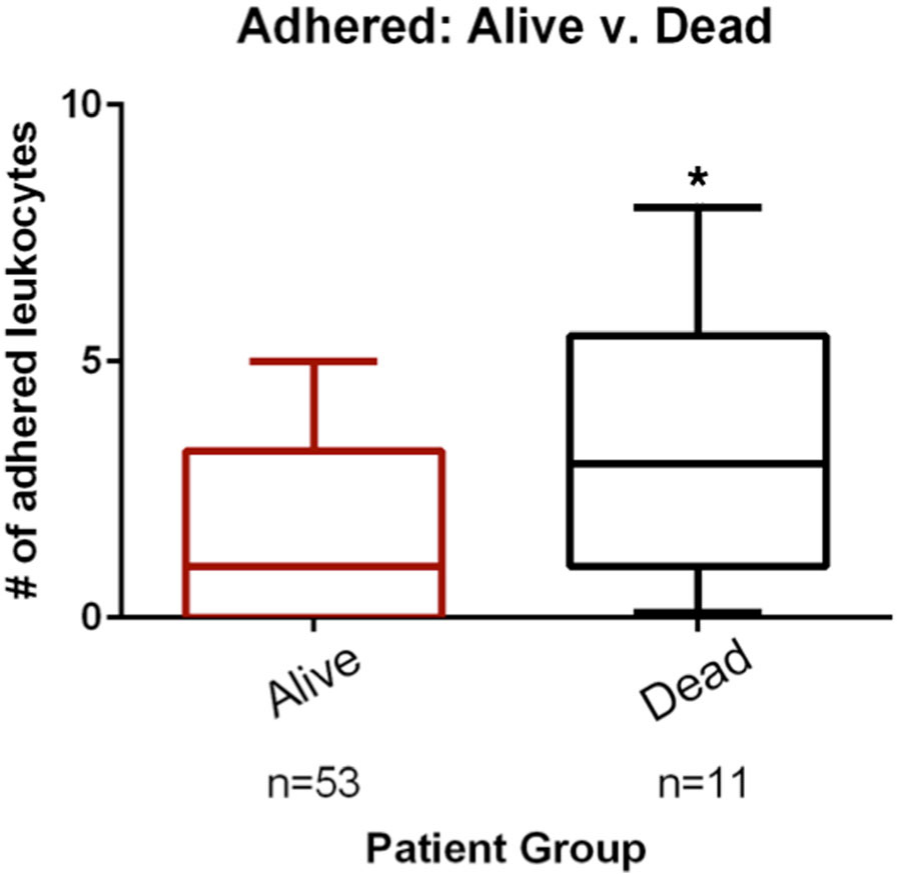
Comparison of rolling leukocytes in patients with sepsis and in controls. The boxes delineate the interquartile range, the median is shown as a line in the middle of the box, and tails show the 95% range; ***p < 0.001

There was an increase in the median number of adhered leukocytes per field in the sepsis group 1.0 (IQR 0–3.5) compared to 0 (0–0) in the non-infected group (*p* < 0.001) (Fig. 2). This corresponded to a median number of adhered leukocytes per unit vessel length of 0.07/mm (0–0.23) and 0.03/mm (0–0) for sepsis and non-infected groups, respectively (*p* < 0.001). For the rolling leukocytes, we observed a median of 26 (10.3–42) in the sepsis group and 9.8 (4.8–17.3) in the non-infected group (*p* < 0.001) per field (Fig. 3). This corresponded to a median number of rolling leukocytes per unit vessel length of 1.7 (0.7–2.7) and 0.6/mm (0.4–1.0), respectively (*p* < 0.001). We also performed a sensitivity analysis by adjusting by perfused vascular density (PVD) and total vascular density (TVD), and found no meaningful differences in the results.

### Leukocyte rolling and adhesion in sepsis survivors versus non-survivors

**Fig. 4:**
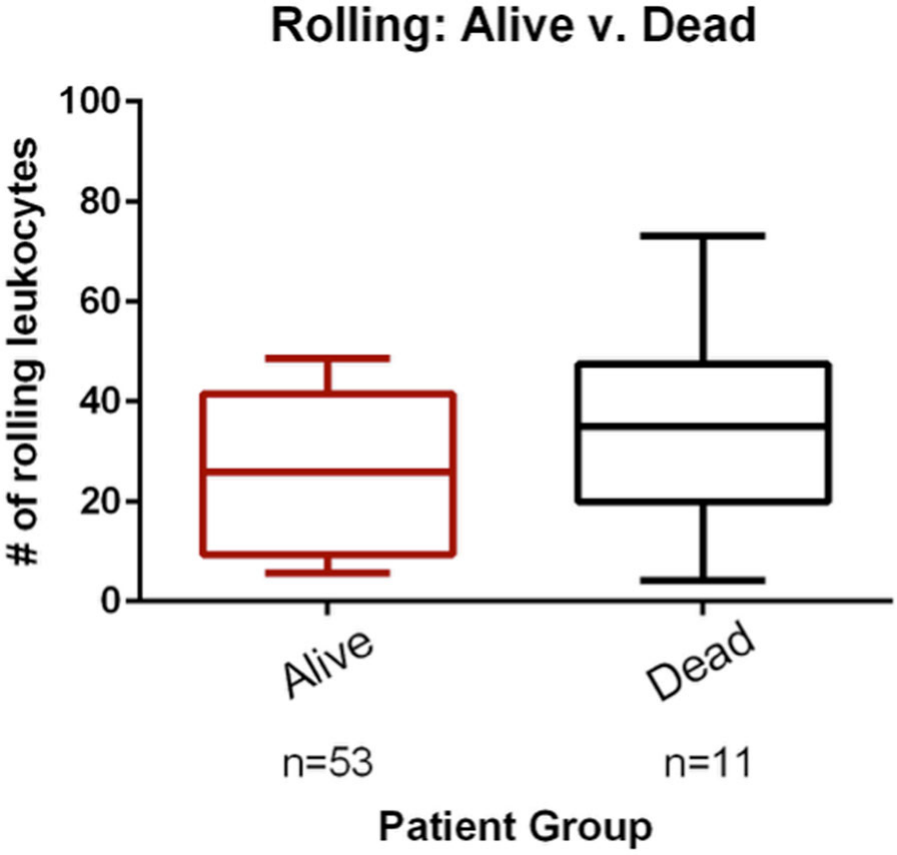
Comparison of adhered leukocytes in survivors and non-survivors in the sepsis group. The boxes delineate the interquartile range, the median is shown as a line in the middle of the box, and tails show the 95% range; *p < 0.05

**Fig. 5:**
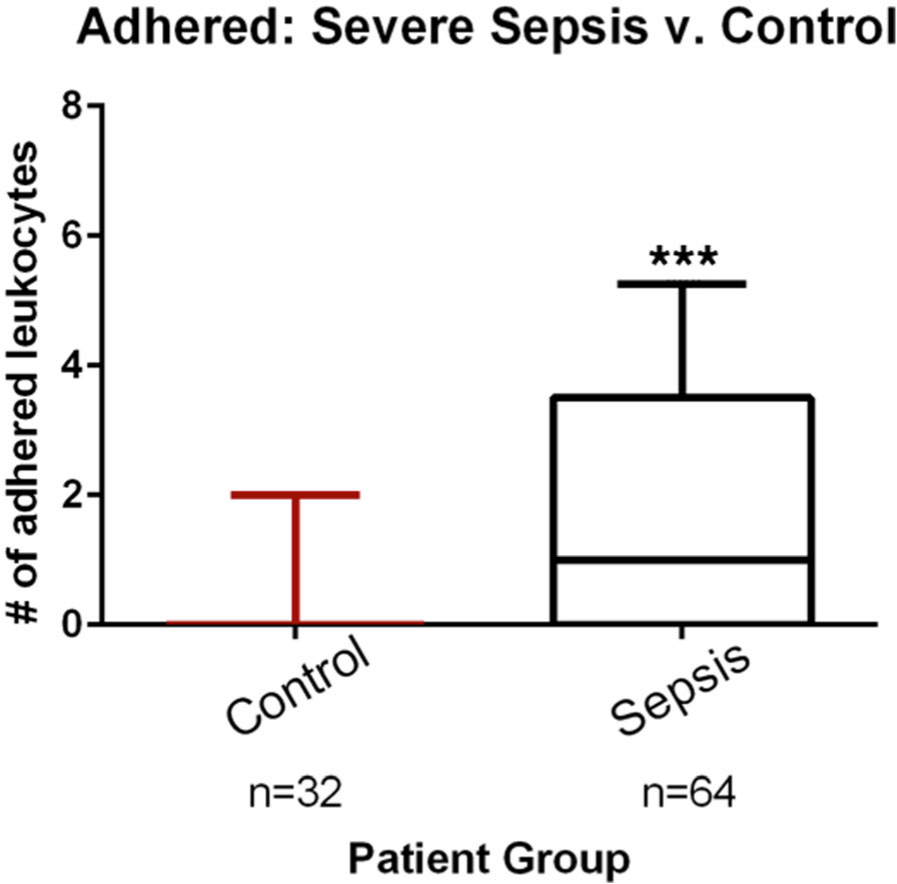
Comparison of rolling leukocytes in survivors and non-survivors in the sepsis group. The boxes delineate the interquartile range, the median is shown as a line in the middle of the box, and tails show the 95% range. The groups were not significantly different, *p* = 0.31

Among the patients with sepsis (*n* = 64), there was an increase in the number of adhered leukocytes in non-survivors compared to survivors (3.0 (1–5.5) vs. 1.0 (0–3.0), *p* < 0.05) (Fig. 4); this corresponds to a vessel-length adjusted value of 0.17 (0.07–0.31) vs. 0.07 (0–0.22), *p* = 0.07. However, there was no difference in rolling leukocytes (35 (20–48) vs. 26 (10–41), *p* = 0.31); vessel-length adjusted value of 2.0 (1.2–4.1) vs. 1.7 (0.6–2.5), *p* = 0.35 (Fig. 5). Here, we also performed a sensitivity analysis by adjusting by PVD and TVD, and found no meaningful differences in the results.

## Discussion

Our findings are initial evidence in support of the use of the sublingual mucosa as a window for in vivo human studies of leukocyte activation in sepsis as sublingual leukocyte rolling and adherence may hold potential as a diagnostic and prognostic marker. These data provide support for further investigation in this domain.

In prior studies with orthogonal polarization spectral (OPS) and SDF imaging of the human sublingual mucosa rolling leukocytes were generally defined as leukocytes moving at a slower velocity than the column of erythrocytes [10–12]. This is similar to the definitions used in studies utilizing intravital microscopy. Thus, rolling leukocytes are identified as slow-moving leukocytes moving along the endothelial surface with temporary low-affinity bindings that are continuously overcome by the shear forces of the main blood flow, resulting in the characteristic rolling motion. Also, rolling leukocytes can detach from the endothelial cells and go back into the main blood stream. We regarded all leukocytes moving slower, even stopping intermittently, or detaching back into the main bloodstream as rolling. In intravital studies leukocyte adherence is defined as leukocytes sticking to the endothelium for a given time; however the time often varies. Definitions vary between 1 and 60 s (even 10 min), with > 30 s being the most common, which correlates to the time that a leukocyte needs to stay firmly adhered to initiate extravasation [2]. Observations in the human sublingual area of a single vessel segment over a long period of time are very difficult. We defined adhered leukocytes as leukocytes that started and remained in the same place on the endothelium throughout the video (3 s).

Previous studies with OPS imaging, the predecessor of SDF imaging, assessed sublingual leukocyte activation in humans during cardiopulmonary bypass and in humans exposed to high altitude [1, 10, 11, 20]. Despite no alterations in microcirculatory flow velocity, a significant increase in activated (rolling) leukocytes observed sublingually during cardiopulmonary bypass along with significant correlation with CD18 expression (and systemic white blood cell (WBC) count) was seen. Only a moderate decrease in functional capillary density was seen. Thus, the authors found evidence of a systemic inflammatory reaction to cardiopulmonary bypass, which could be directly visualized sublingually independently of microcirculatory flow velocity [11]. This supports the hypothesis that the sublingual area can be used as a surrogate window to visualize systemic inflammatory reactions. In a study looking at the sublingual microcirculation during exposure to high altitude, there were no observed differences in leukocyte rolling or microcirculatory flow [10].

In an investigation similar to ours, Donati et al. quantified activated leukocytes along with measurements of glycocalyx degradation in critically ill patients and healthy control patients using SDF imaging [12]. They found significant correlation between glycocalyx degradation and number of rolling leukocytes, suggesting that leukocyte activation and endothelial injury may be assessed through the sublingual mucosa as an early diagnostic modality in sepsis [12]. Donati et al. compared the sublingual vascular glycocalyx between critically ill ICU patients and healthy volunteers. The perfused boundary region (PBR), which is proposed as a parameter inversely related to glycocalyx thickness, was increased in ICU patients compared to controls and most profound among ICU patients with sepsis. Donati et al. found a positive relationship between rolling leukocytes and PBR (presumed indicator of thinned glycocalyx) [12]. This adds evidence in support of the proposition that leukocyte activation and endothelial injury may be assessed through the sublingual mucosa in patients with systemic inflammation - in this case early septic shock.

Future directions include refinement, standardization, and/or automation of techniques to quantify the presence of rolling and adhered leukocytes. Alternative methods may utilize approaches used in intravital microscopy, e.g. measuring a rolling flux of leukocytes (count of rolling leukocytes crossing a designated perpendicular line) per time unit, or simply restrict the analysis to a fixed number of vessels. Another approach could be the subdividing of the FOV, so that investigators do not have to keep track of activated (rolling) leukocytes in the whole FOV [10, 11]. Also, as the resolution of videomicroscopy imaging devices improves, visualization may be more accurate.

### Limitations of the study

We lack an available automated technique for quantification of rolling and adhered leukocytes. We relied on manual identification of rolling and adhered leukocytes, which is prone to human error or imprecision, leaving us susceptible to misclassification bias. Another limitation is our convenience samples of patients and controls, leaving us prone to selection bias. In addition to that, we excluded videos if they did not conform to certain quality criteria.

A previous study showed that hemorrhage in rats reduced shear rate resulting in increased leukocyte-endothelial cell adhesion [21]. However, whether this is a physical phenomenon or a molecular one is not clearly delineated. We did not have hemodynamic data available at the time of image acquisition so we were unable to consider the influence of these measures on rolling and adhesion. We also did not stratify by vessel diameter, observing leukocytes primarily confined to post-capillary venules with a diameter greater than 20 μm; it is possible that discriminating by size or type of vessel may yield different numbers. Although less time consuming compared to the traditional manual analysis of microcirculatory flow parameters, leukocyte quantification is still quite time consuming - especially when counting in the whole field of view.

## Conclusions

We demonstrated the physiologic expression of endothelial cell activation manifested by a significantly higher number of rolling and adhered leukocytes in patients with septic shock as compared to non-infected control patients. In the sepsis group there were more rolling and adhered leukocytes as compared to non-infected control patients. In the sepsis group, there were more adhered leukocytes in non-survivors compared to survivors. The sublingual mucosa may be a window to an important physiologic manifestation of leukocyte and endothelial cell activation in sepsis.

## Additional file

Additional file 1: Videomicroscopy Movie of Adhered Leukocytes. (DOCX 42 kb)

## Abbreviations

ED: Emergency department
FDA: Food and Drug Administration
FOV: Field of view
ICU: Intensive care unit
LED: Light-emitting diode
OPS: Orthogonal polarization spectral
PBR: Perfused boundary region
ProCESS: Protocolized Care for Early Septic Shock
SD: Standard deviation
SDF: Sidestream dark field
SIRS: Systemic inflammatory response syndrome
WBC: White blood cell count
