# Supplementary material for: In vivo quantification of rolling and adhered leukocytes in human sepsis
Source: Crit Care. 2018 Sep 30;22:240. doi: 10.1186/s13054-018-2173-z (PMC6164176; doi:10.1186/s13054-018-2173-z)
Supplement: Supplementary file 1 — Videomicroscopy Movie of Adhered Leukocytes. (DOCX 42 kb) [file 13054_2018_2173_MOESM1_ESM.docx]

**Uncompressed MHA Video Preprocessing in Matlab**

PreprocessMHA(mhafile,outdir,dofilter)

Raw, uncompressed MHA video frames were filtered using a 5 by 5 pixel Gaussian kernel followed by linear contrast stretching and an adaptive histogram equalization. Finally, the filter cascade output was downsampled by a factor of 3 to 736 (width) by 549 (height) pixels, to approximately fit the maximum display frame size in AVA 3.2 software (Automated Vascular Analysis (AVA), MicroVision Medical BV, Amsterdam, The Netherlands). The output pixel pitch was 1.98 µm (square pixels).

**Feature Based video Stabilization in Matlab**

We implemented a novel video stabilization method that presumes that small vessels are located in the plane of focus and that we only seek to correct for camera motion in the plane of focus. The specific method is beyond the scope of this report.

Method: Preprocessed video frames (736 x 549 pixels as before) were processed with a multiscale Frangi filter. The output of the Frangi filter represents a vessel probability density for each pixel. The output is image based, so further processing is required to extract vessel centerlines and widths. Other methods are preferred for vessel centerline and lumen measurements. The strongest features are automatically extracted for tracking the camera motion in the field of view. Stabilized output video is rendered from the estimated camera motion.

**Temporal Smoothing**

A kernel of stabilized frames was averaged over time with a Gaussian weighting. The width of the Gaussian corresponds to a synthetic exposure time for each video frame. Each frame of video corresponds to 1/25 s. The temporal average filter had a width of approximately 1.5 frames, corresponding to a synthetic exposure time of 0.06 s.

**Frame Averaging for Adhered Leukocyte Detection**

Video files were stabilized in order to minimize motion artifacts. Frame averaging introduces a persistence effect with potential for image blurring if the camera or tissue moves too rapidly between frames. Frame averaging is used with relative large kernels (>16 frames) in AVA and CytoCamTools Analysis Software (Braedius Medical B.V., Huizen, The Netherlands) in order to fill in plasma gaps and smooth out vessel profiles for automated and manual vessel detection and outlining. For our purposes, it is sufficient to blur the fast-moving erythrocytes while keeping the brighter and slower moving activated leukocytes sharp.

Rather than analyzing a static output image, we process every frame of video with our frame averaging algorithm and rely on an image analyst to identify the enhanced leukocytes as they roll along the vessel lumen.

We used a Gaussian kernel *h_g_(n)*with a half width, σ equal to one (frame) where:

$$h_{g}\left( n \right)=e^{-\frac{n^{2}}{2\sigma^{2}}}$$

And $h\left( n \right)=\frac{h_{g}\left( n \right)}{\sum_{m} h_{g}\left( m \right)}$

The Gaussian kernel was convolved with the video sequence and rendered at different frame rates to assist the analyst in viewing the enhanced leukocyte motion.

**Discussion**

We assume a Gaussian intensity profile for the leukocyte with full width at half maximumequal to the width of a neutrophil in suspension, approximately 9 µm[1]. Note that adhered leukocytes will have diameters that are approximately 10-15% larger[2]. In order for the intensity of a moving leukocyte to add constructively (and be indistinguishable) in two consecutive (averaged) frames, the separation between the two leukocytes must be less than the half-width or σ of the Gaussian intensity profile. Thus, if the leukocyte motion between two consecutive frames is less than or equal to approximately 4.5 µm, the leukocyte intensity will be preserved.


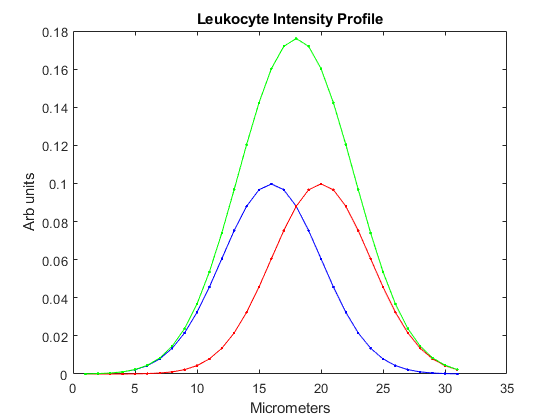


Figure. Leukocyte intensity profile. Blue and Red intensity profiles are shifted by σ, the half-width of the Gaussian intensity profile. If the leukocyte motion is less than σ per video frame, then the resultant intensity will add maximally.

Frame averaging pseudo code:

Let the input frame be *I(n)*, the output frame be *J(n)*, the total number of input frames is *N,* and the number of (odd) filter elements are *k.*

For each input frame *I(n)* and output frame *J(n)*:

For each index *i* of the filter kernel from 1 to *k*:

*J(n)* := *h(i)* * *I(n – k/2 + (i-1) * (k/2))*

Note that the output frames numbered 1 to *k/2* and *N* – *k/2* to *N* are not defined and must be handled as special cases or dropped. In our case, σ = 1, and the kernel falls off to zero rapidly for *k* >= 5. For example, the kernel for *k=*3 is [0.2741, 0.4519, 0.2741] and, for *k*=5, [0.0545, 0.2442, 0.4026, 0.2442, 0.0545].

**References:**

1. Niemiec MJ, De Samber B, Garrevoet J, Vergucht E, Vekemans B, De Rycke R, Bjorn E, Sandblad L, Wellenreuther G, Falkenberg G *et al*: **Trace element landscape of resting and activated human neutrophils on the sub-micrometer level**. *Metallomics* 2015, **7**(6):996-1010.

2. Roca-Cusachs P, Almendros I, Sunyer R, Gavara N, Farre R, Navajas D: **Rheology of passive and adhesion-activated neutrophils probed by atomic force microscopy**. *Biophys J* 2006, **91**(9):3508-3518.
